# MicroRNA regulation of cancer stem cells in the pathogenesis of breast cancer

**DOI:** 10.1186/s12935-020-01716-8

**Published:** 2021-01-07

**Authors:** Tong Niu, Weiwei Zhang, Wei Xiao

**Affiliations:** 1 Beijing Key Laboratory of DNA Damage Responses and College of Life Sciences, Capital Normal University, Beijing, 100048 China; 2 Department of Biochemistry, Microbiology and Immunology, University of Saskatchewan, Saskatoon, SK S7N 5E5 Canada

**Keywords:** Breast cancer, Breast cancer stem cell (BCSC), miRNA, Self-renewal, Therapeutic resistance

## Abstract

Breast cancer is the most common cancer among women and accounts for 30% of all female malignancies worldwide. Breast cancer stem cells (BCSCs) are a small population of breast cancer cells that exhibit multiple characteristics including differentiation capacity, self-renewal and therapeutic resistance. Recently, BCSCs have attracted attention due to their modulation of breast tumor behaviors and drug resistance. miRNAs are small noncoding mRNAs involved in virtually all biological processes, including stem cell development, maintenance and differentiation. In breast cancer, miRNAs appear to be multi-faceted since they can act as either suppressors or oncogenes to regulate breast cancer progression. This review summarizes the critical roles of miRNAs in regulating multiple signaling pathways such as Wnt/β-catenin, Notch, PI3K/AKT/mTOR, BMI-1 and STAT3 that are important for the BCSC maintenance.

## Background

Breast cancer (BC) is the most common female cancer in terms of high incidence and mortality rate all over the world. The prevalence of BC is increasing to estimated 42,690 deaths and 279,100 new cases in the United States in 2020 (10.3322/caac.21590). In women, BC is the most common type of cancer (25%) and the leading cause of cancer deaths (15%) [1]. High hormonal status, such as estrogen, estrogen receptor (ER), progesterone receptor (PR) and human epidermal growth factor receptor (HER2), are the most important factors promoting BC onset and progression. Four distinct breast cancer subtypes have been classified based on the expression of the proliferation marker (Ki67) and the hormonal status including Luminal A, Luminal B (HER2 negative), Luminal B (HER2 positive), HER2 (non-luminal) and triple-negative breast cancer (TNBC) [2] (Table 1).

**Table 1:** The subtypes of breast cancer classified via expression of Ki67 and hormonal status

Subtypes	The expression of Ki67 and hormonal status
Luminal A	Estrogen positive (ER** +**)
	Progesterone receptor (PR) ≥ 20%
	HER2 negative (HER2-)
	Ki67 < 14%
Luminal B (HER2−)	Estrogen positive (ER** +**)
	HER2 negative (HER2-)
	Ki67 ≥ 20%
	Progesterone receptor (PR) < 20% or progesterone receptor negative (PR-)
Luminal B (HER2+)	Any progesterone receptor (PR) level
	Any Ki67 level
	HER2 positive (HER2+)
	Estrogen positive (ER+)
HER2 positive (HER2+) (non-luminal)	HER2 positive (HER2+)
	Estrogen negative (ER-)
	Progesterone negative (PR−)
Triple-negative breast cancer (TNBC)	HER2 negative (HER2−)
	Estrogen negative (ER−)
	Progesterone negative (PR−)

Many clinical and pathological factors determine the prognosis of BC. In the past decades, a number of cellular factors such as onco-proteins, circulating tumor cells, mutations in some specific genes, microRNAs (miRNAs) and cancer stem cells (CSCs) were proposed to be potential parameters for the prognosis of breast cancers [3]. CSCs in BC tissues lead to uncertainty for breast cancer treatment. CSCs were first detected in human acute myeloid leukemia (AML) and they are highly malignant, as they facilitate tumor metastasis, relapse, tumor progression and confer resistance to cancer therapy. Besides the common cancer cell capability such as migration and invasion, these cells exhibit characteristic stem cell properties including differentiation, self-renewal and tumor-initiation [4]. Increasing evidence shows that CSCs are closely associated with the pathogenesis and progression of various cancers, such as pancreatic, colon, prostate, brain and breast cancers. They could confer resistance to therapeutic agents and contribute to the propagation of neoplastic cells for tumor heterogeneity [5]. Somatic and normal stem cells could serve as sources of CSCs under abnormal genetic and epigenetic changes.

miRNAs are small non-coding RNAs with a length of 21–25 nucleotides which are known to regulate more than 60% of human genes at the post-transcriptional level by targeting 3’-untranslated regions (3′‐UTR) of mRNAs for inactivation or degradation. miRNAs play vital roles in multiple biological processes, such as development, cell proliferation and apoptosis. Aberrant expression of miRNAs is closely related with CSC maintenance and tumorigenesis [4]. This review focuses on roles of miRNAs in breast cancer progression, particularly their involvement in Wnt/β-catenin, Notch, PI3K/AKT/mTOR, BMI-1 and STAT3 pathways in breast cancer stem cells (BCSCs).

### Breast cancer stem cells

BCSCs contribute to breast tumor initiation, malignancy and therapeutic resistance. They confer capability in differentiation and self-renewal [6, 7]. Although they only account for approximately 2% breast tumor cells, BCSCs are mainly responsible for metastatic growth, high morbidity and mortality of BC. In addition, BCSCs could result in recurrence and relapse of BC due to their resistance to therapeutic treatments [8]. So far, despite the controversy, it is suggested that BCSCs originate from somatic and normal stem cells [9]. Several BCSC-specific biomarkers including CD44, CD24, aldehyde dehydrogenase (ALDH1) [10], epithelial specific antigen (ESA), CD326 (EpCAM) [11], CD133 [12], CD61 and CD49f [13] have been identified. These biomarkers are vital for BCSC isolation and potentially serve as therapeutic targets for BC treatment.

### miRNA regulation of CSCs in breast cancer

**Fig. 1:**
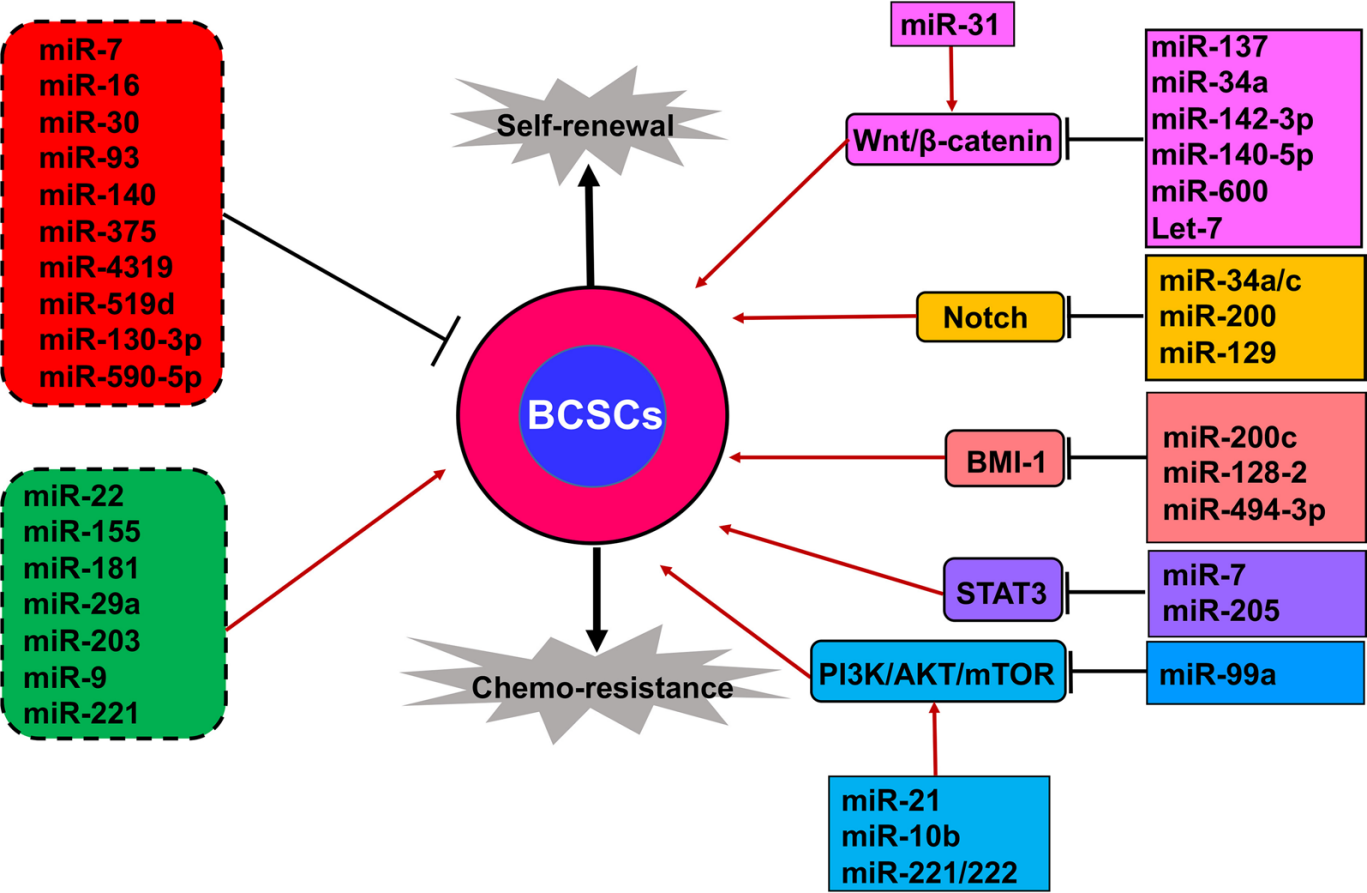
miRNAs act as suppressors or oncomiRs in BCSC maintenance. miRNAs can both act as suppressors (red background) or oncomiRs (green background) to influence BCSC activities independently of known signaling pathways (left panel). On the other hand, some miRNAs can be placed into well-defined signaling pathways (right panel, different color backgrounds) by positively or negatively regulating the characteristics and development of BCSCs such as self-renewal or chemoresistance capacities

As mentioned above, miRNAs play critical roles in CSC differentiation and self-renewal. Emerging evidence suggests that miRNAs are also promising targets and powerful tools for therapeutic treatments of breast cancer via regulation of BCSC differentiation and self-renewal [1]. Here, we dissect and summarize the roles of BCSCs-related miRNAs during breast cancer progression (Fig. 1).

### **miRNAs act as breast cancer suppressors** (Table 2)

**Table 2:** miRNA acts as suppressor in breast cancer

miRNA	Target	Result on BCSCs
miR-7	KLF4	Inhibits self-renewal, pluripotent potential metastatic and invasive ability of BCSCs
miR-93	HMGA2	
	AKT3	
	EZH1	Suppresses EMT and metastasis
	JAK1	Inhibits differentiation and proliferation of BCSCs
	SOX4	
	STAT3	
miR-30	UBC9	Inhibits self-renewal ability of BCSCs
	ITGB3	Induces apoptosis of BCSCs
miR-590-5p	SOX2	Inhibits stemness, metastasis, population and tumorigenicity of BCSCs
miR-140	ALDH1	Reduces stemness phenotypes of BCSCs
	SOX9	Inhibits tumor growth and formation in vivo
miR-16	Wip1	Inhibits growth and self-renewal capacity of BCSCs
miR-130-3p	RAB5B	Represses proliferation, migration and invasion of BCSCs
miR-4319	E2F2	Inhibits tumorigenicity and self-renewal of BCSCs
miR-519d	MCL-1	Suppresses BCSCs’ chemoresistance to cisplatin
miR-375	HOXB3	Inhibits stemness phenotypes, EMT, migration and invasion of BCSCs

#### miR-7

miR-7 is expressed poorly in CD24^−^/CD44^+^/ESA^+^ BCSCs with potency to metastasize to brain and bone. It represses the brain but not bone metastasis of BCSCs by inversely regulating the expression of pluripotency marker *KLF4* [14, 15]. Experiments performed in vitro revealed that miR-7 decreases BCSC’s self-renewal and invasive ability by inhibiting the expression of *KLF4* [14]. These results indicate that miR-7 and KLF4 may act as promising therapeutic targets and biomarkers for brain metastasis of BC.

#### miR-93

miR-93 belongs to the miR106b-25 cluster and may function as a tumor suppressor, although it is frequently overexpressed in various human malignant cancers [16, 17]. Expression of miR-93 downregulates multiple stem cell regulatory genes in BCSCs, including *HMGA2*, *AKT3*, *EZH1*, *JAK1*, *SOX4* and *STAT3*, and ultimately leads to Mesenchymal-Epithelial Transition (MET). Mouse xenograft models reveal that ectopic expression of miR-93 completely blocked the metastatic and tumor development abilities of BC. In addition, experiments with different breast cancer cell lines representing various differentiation states and mouse xenograft models reveal that miR-93 expression is lower in most undifferentiated and basal breast cancer cells, and that overexpression of miR-93 increases CSC population and tumor growth in more differentiated luminal breast cancer cells. These results suggest that miR-93 regulates the fate of BCSCs via controlling their differentiation and proliferation states [18].

#### miR-30

The miR-30 level is decreased in BCSCs, which is accompanied by increased expression of its target genes *ITGB3* and *UBC9*. Furthermore, miR-30 inhibits self-renewal and induces apoptosis of the BCSCs mainly through repressing these two targets. Moreover, a study using a BCSC xenograft mouse model demonstrates that overexpressed miR-30 reduces lung metastasis and tumorigenesis [19]. In addition, miR-30 modulates non-attachment growth and the expression of proliferation and apoptosis related genes in putative BCSCs, and is negatively associated with BC progression [20]. These findings suggest that miR-30 regulates the stemness of BCSCs to repress tumorigenesis and metastasis of BC.

#### miR-590-5p

miR-590-5p is a potential target for breast cancer therapeutic treatments. It significantly reduces the population of BCSCs. In vivo NOD/SCID nude mice experiments reveal that miR-590-5p significantly inhibits tumorigenicity of BCs [21]. At the molecular level, miR-590-5p is able to repress the expression of a key stemness marker *SOX2* and thus inhibits BCSCs’ stemness and metastasis.

#### miR-140

Ductal carcinoma in situ (DCIS) is an early stage of BC and reducing the incidence of DCIS is the major goal of breast cancer prevention [22]. Volinia et al. found that miR-140 regulates BCSCs in luminal subtype invasive ductal carcinoma [23]. Li et al. demonstrated that loss of miR-140 is a hallmark of DCIS lesions and that miR-140 is significantly downregulated in BCSCs. They also found that restoration of miR-140 could decrease the expression of stem cell marker *ALDH1* and *SOX9*, and reduce basal-like breast tumor growth in vivo [24]. These results suggest that miR-140 is related to maintenance of basal-like DCIS CSCs and deregulated stem cell signaling. Furthermore, miR-140/SOX9/ALDH1 axis is important for basal-like breast tumor formation, BCSCs’ self-renewal and a potential therapy target for basal-like DCIS patients [24].

#### miR-16

To uncover the biological functions of miR-16 in the progression of breast cancer, Zhang et al. examined its expression in various BCs and BCSCs, and found that miR-16 is significantly downregulated in BCSCs. Moreover, elevated expression of miR-16 inhibits the growth and self-renewal capacity of mouse mammary stem cells by inhibiting its target gene *Wip1.* More importantly, it confers MCF7 BC cells increased sensitivity to the chemotherapeutic drug doxorubicin [25].

#### miR-130-3p

miR-130-3p is downregulated in breast cancer cells and tissues. Overexpression of miR-130-3p in BCSCs inhibits cell proliferation by inducing G0/G1 arrest. Its elevation also represses BC cell migration and invasion by directly downregulating the expression of the oncogene *RAB5B* [26]. Consistently, miR-130-3p depletion exhibits the opposite effects [26].

#### miR-4319

Upregulated miR-4319 markedly reduces the stemness and tumorigenicity of stem cells in triple-negative breast cancer (TNBC) via suppressing the expression of *E2F2*, a transcription factor vital for stem cell self-renewal, while downregulation of miR-4319 promotes tumorsphere formation and self-renewal in TNBC CSCs and also promotes tumor initiation and metastasis in vivo [27].

#### miR-519d

miR-519d was found to be downregulated in BCSCs. The experimental expression of miR-519d enhances BCSCs’ sensitivity to the chemotherapeutic drug cisplatin through an MCL-1 (an anti-apoptotic protein)-dependent mitochondria pathway [28]. Therefore, miR-519d could serve as a breast tumor suppressor by reducing chemoresistance in BCSCs.

#### miR-375

The stemness of MCF7 cells can be suppressed by miR-375. miR-375 also inhibited EMT, cell migration and invasion as well as tamoxifen-resistance by degrading its direct target *HOXB3* in human ER-positive breast cancers [29], indicating that targeting miR-375 and HOXB3 can be a promising therapeutic method for ER-positive breast cancer patients.

### **miRNAs act as breast cancer oncogenes** (Table 3)

**Table 3:** miRNA acts as oncomiR in breast cancer

miRNA	Target	Result on BCSCs
miR-155	CD44	Enhances BCSCs’ chemoresistance to doxorubicin
	CD90	
	ABCG2	Promotes BCSCs’ formation
miR-181	BRCA1	Increases stemness, colony formation and phenotypes of BCSCs
miR-22	TET family	Increases stemness and metastasis of BCSCs
miR-29a	H4K20	Promotes metastasis and invasion of BCSCs
miR-9 and miR221	CD133	Enhances stemness, proportion and tumor sphere formation of BCSCs
	Nanog	
	Oct4	
miR-203	SOCS3	Promotes proliferation, growth and self-renewal of BCSCs

#### miR-155

miR-155, also known as B-cell integration cluster (BIC), was first identified in B-cell lymphomas [30]. miR-155 is commonly overexpressed in multiple solid malignancies besides breast cancer [31]. Inhibition of miR-155 markedly suppresses the formation of BCSCs by repressing the expression of stem cell markers CD44, CD90 and ABCG2. In addition, inhibition of miR-155 decreases proliferation of breast cancer cells and sensitizes MDA-MB-231 BC cells to the chemotherapeutic drug Doxorubicinol. Taken together, miR-155 may be an oncomiR and a promising therapeutic target of breast cancer; it is also closely associated with sensitivity to chemotherapy and BCSC formation [31, 32].

#### miR-181

miR-181 has been reported as an oncogenic miRNA. It promotes colony formation, self-renewal capacity and tumor development in breast cancer [5]. Transforming growth factor-β (TGF-β) regulates the sphere-forming stem cell-like feature of BCSCs by upregulating miR-181 and downregulating ATM [33]. TGF-β induces impaired DNA-repair efficiency and synthetic lethality to the inhibition of PARP by downregulating the DNA-repair gene *BRCA1* through an miR-181-mediated mechanism in breast cancer. Furthermore, the miR181/BRCA1 axis plays a vital role in primary breast tumor by promoting CSC phenotypes [1, 34].

#### miR-22

Genetic and epigenetic alterations including altered miRNA expression are largely responsible for distant-organ metastasis of tumor cells. miR-22 promotes breast tumor metastasis via Ten eleven translocation (TET) family-dependent chromatin remodeling of *miR-200* and it eventually inhibited miR-200 activity [35]. miR-22 could expand the stem cell compartment and enhance mammary gland side-branching in a mammary gland-specific transgenic mouse model [35].

#### miR-29a

It was recently reported that miR-29a is increased in breast cancer tissues and BC cells like MCF7 and BCSCs [36]. miR-29a markedly inhibits the expression of *SUV420H2*, encoding a histone methyltransferase that specifically trimethylates H4K20, via down-regulating histone H4K20 trimethylation and thus promotes BCSC metastatic capacities [36]. Moreover, miR-29a promotes breast cancer EMT via attenuating the repression of connective tissue growth factor (CTGF) and early growth response protein 1 (EGR1) by H4K20 trimethylation [36]. These findings collectively suggest that miR-29a is an oncomiR and is central for breast cancer EMT and metastasis via targeting BCSCs.

#### miR-9 and miR-221

It has been observed that overexpression of miR-9 and miR-221 dramatically increased BCSCs stemness, migration and invasion via increasing the number of side-population colonies with stem cell-like potency. Consistently, inhibition of miR-9 and miR-221 reduced the proportion and tumor-sphere formation of BCSCs by reducing the expression of the stemness markers *Nanog, CD133* and *Oct4*. Moreover, increased levels of miR-9 and miR-221 in BC are closely related to elevated risk of progression to malignancy, poor differentiation, lymph-node metastasis, reduced survival, late-stage evolution and increased tumor size [37]. Hence, miR-9 and miR-221 have tumorigenic capacity as they promote BCSCs’ properties to yield an invasive phenotype in BC.

#### miR-203

Upregulated expression of miR-203 was detected both in ER-positive (ER^**+**^) BC cell lines and BC tissues. Inhibition of miR-203 represses ER^+^ breast cancer proliferation, growth and self-renewal capacity of BCSCs by negatively regulating suppressor of cytokine signaling 3 (SOCS3) expression [38]. These findings suggest that miR-203 serves as an oncomiR and may be a useful therapeutic target for ER^+^ BC treatment.

### miRNA regulation of CSCs’ signaling pathways in breast cancer

#### Roles of miRNAs in the Wnt/β-catenin signaling pathway

**Fig. 2:**
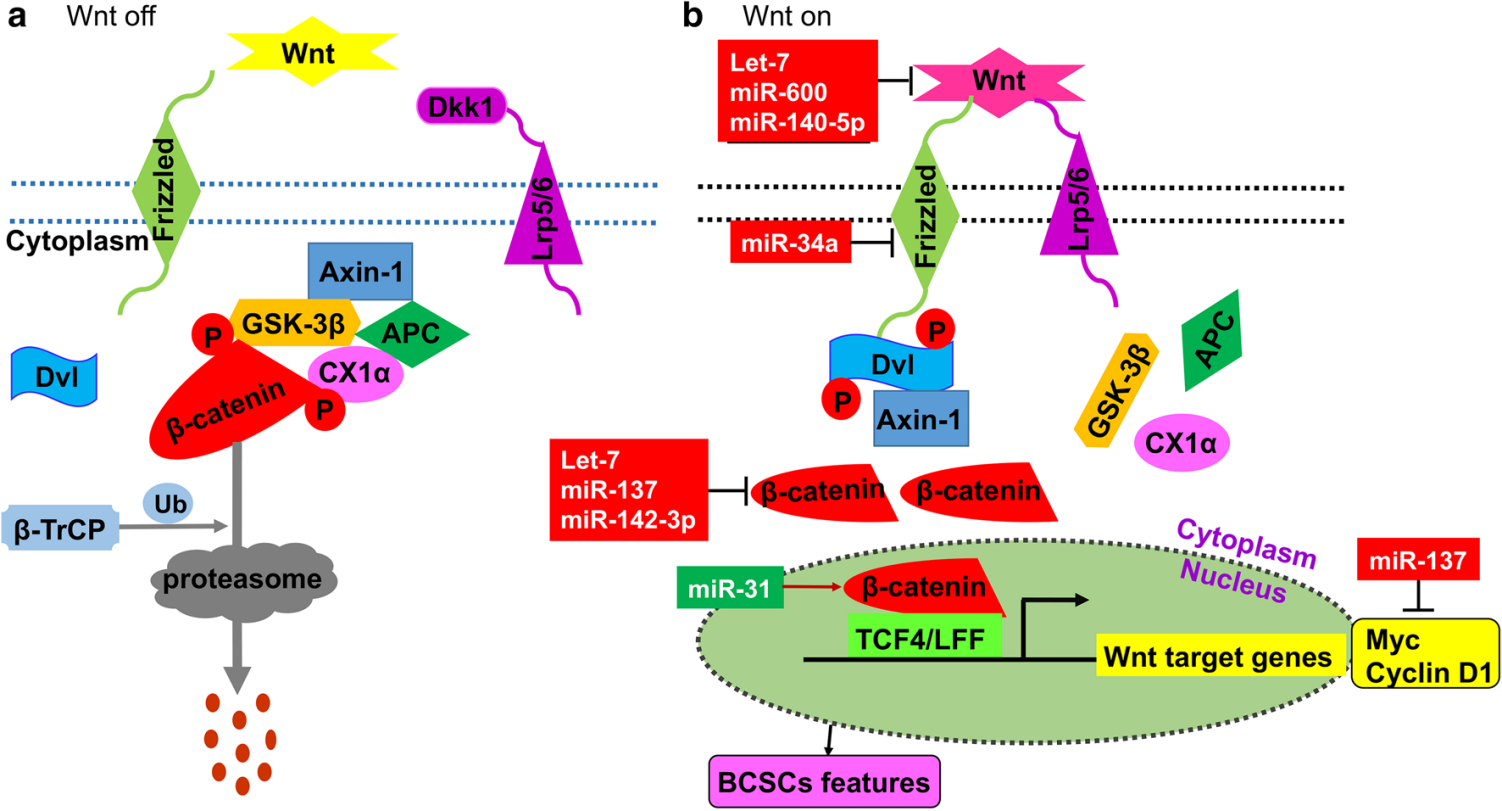
miRNA-mediated regulation of BCSCs via targeting the Wnt/β-catenin signaling pathway. **a** “Wnt off”. In the absence of Wnt ligands, the destruction complex (Axin-1, GSK-3β, APC, CX1α) is formed. β-catenin is phosphorylated by the destruction complex, thereby targeting it to be degraded by the β-TrCP-mediated ubiquitin proteasome system. **b** “Wnt on”. Wnt ligands bind to the Frizzled/Lrp 5/6 receptors, thus leading to Dvl phosphorylation. Phosphorylated Dvl recruits Axin to the membrane, which destroys the destruction complex and prevents the phosphorylation of β-catenin. Thus, β-catenin accumulates in the cytoplasm and finally moves into the nucleus, where it interacts with TCF4/LEF and/or co-activators and promotes the transcription of Wnt target genes. The immediate targets of several miRNAs are marked

The Wnt/β-catenin signaling pathway is important for CSC maintenance and closely associated with development of different cancers [39, 40]. In the canonical Wnt/β-catenin signaling pathway, there are two scenarios, “Wnt off” and “Wnt on.” “Wnt off” refers to the absence of Wnt ligands, in which the destruction complex, consisting of APC (adenomatous polyposis coli), GSK-3β (glycogen synthase kinase 3 beta), Axin-1 and CK1α (casein kinase 1), is formed. This destruction complex modulates the cytoplasmic β-catenin level by phosphorylating β-catenin and targeting it for proteasomal degradation. “Wnt on” refers to the presence of Wnt ligands, which leads to Dvl (disheveled protein) phosphorylation via Wnt ligands binding to Frizzled/low density lipoprotein receptor-related proteins 5 or 6 (Lrp5/6). Dvl recruits Axin-1 to disrupt the destruction complex and stabilize β-catenin in the cytoplasm, which is translocated to the nucleus and binds to TCF4/LEF family transcription factors or other co-activators to activate target genes [41, 42] (Fig. 2). The Wnt pathway is highly activated in many type of CSCs [43].

It has been reported that some miRNAs regulate BCSC activities via the Wnt/β-catenin pathway [44, 45] (Fig. 2), and the majority act as tumor suppressors. For example, FSTL1 (Follistatin Like 1) could increase breast cancer proliferation and stem cell marker expression, and it is closely associated with doxorubicin (DOX) and cisplatin (CDP) chemoresistance in breast cancer cells. Moreover, TOP-flash Wnt signaling luciferase activity assays confirmed that FSTL1 activates Wnt/β-catenin signaling through integrin β3 [46]. Furthermore, it was found that miR-137 directly targeted FSTL1 and reduced its mRNA and protein levels. Ultimately, their findings indicated that miR-137 inhibited the BCSCs’ stemness and chemoresistance via inhibiting the Wnt β-catenin signaling pathway by directly downregulating FSTL1 expression [46].

miR-34a has potential to limit BCSC pools, and is critically associated with growth and maintenance of BCSCs and mammary gland stem cells by targeting the Wnt/β-catenin signaling pathway. For instance, miR-34a limits BCSCs self-renewal capacity by inhibiting the expression of several mesenchymal/basal BCSC markers like N-cadherin, vimentin, ITGA6 and keratin 5, and increasing the expression of CD24 (a marker of luminal differentiation) via inhibiting the Wnt/β-catenin signaling through inhibiting multiple regulators of this pathway like Fzd1, Fzd2 and Pip5k1α. Thus, it would be an attractive strategy to control breast cancer via eradiation of BCSCs by targeting miR-34a-dependent Wnt/β-catenin signaling [47].

Sun et al. used aldehyde dehydrogenase (ALDH1) sorting and mammosphere formation assays showed that miRNA Let-7 inhibited self-renewal of BCSCs in estrogen (ER)-positive breast cancer by blocking the ER activated Wnt/β-catenin signaling pathway [48]. The results of their current study demonstrated that miRNA Let-7 decreases ratio and the self-renewal ability, contributing to reduced tumor formation capacity of BCSCs, and increased the anticancer functions of tamoxifen by suppressing ER and Wnt/β-catenin signaling [49]. Hence, Let-7 has tumor suppressive functions and could enhance endocrine therapy by modulating the stemness of ER-treated CSCs in breast cancer.

It was reported that overexpressed miR-142-3p is accompanied by decreased expression of stem cell makers CD44, CD133, ALDH1, BRCA2, Bod1 and β-catenin levels in BC. It inhibits mammosphere formation, radiation tolerance and BCSC features of BC cells [50]. Thus, miR-142-3p appears to suppress BCSC characteristics and radioresistance by inhibiting the Wnt/β-catenin pathway.

miR-140-5p is commonly considered a tumor suppressor through interaction with stem cell regulators SOX9, SOX2 and Wnt in early stage breast cancer. Consistently, overexpressed miR-140-5p could decrease BCSC populations and inhibit breast cancer progression via suppressing these pathways [51]. Furthermore, miR-140-5p could inhibit BCSC proliferation, tumor-sphere formation and sensitize BCSCs to doxorubicin by downregulating the Wnt/β-catenin signaling pathway [52].

Overexpression of miR-600 could attenuate BCSCs’ self-renewal ability, decrease tumorigenicity in vivo and promote BCSC differentiation via targeting stearoyl desaturase-1 (SCD1), an enzyme required to produce active, lipid-modified Wnt proteins, by inhibiting the Wnt/β-catenin signaling pathway [53].

It has been demonstrated that miR-31 is upregulated in mammary tumors and mammary stem cells (MaSCs) and can enhance MaSC expansion and mammary epithelial proliferation; knocking out miR-31 inhibited breast tumor growth, decreased BCSC populations, tumor-initiating ability and metastasis to lung by activating Wnt antagonist Dkk1 and eventually suppressing the Wnt/β-catenin signaling pathway [54]. To date, miR-31 is the only reported oncomiR in the Wnt/β-catenin pathway.

### Roles of miRNAs in the Notch signaling pathway

**Fig. 3:**
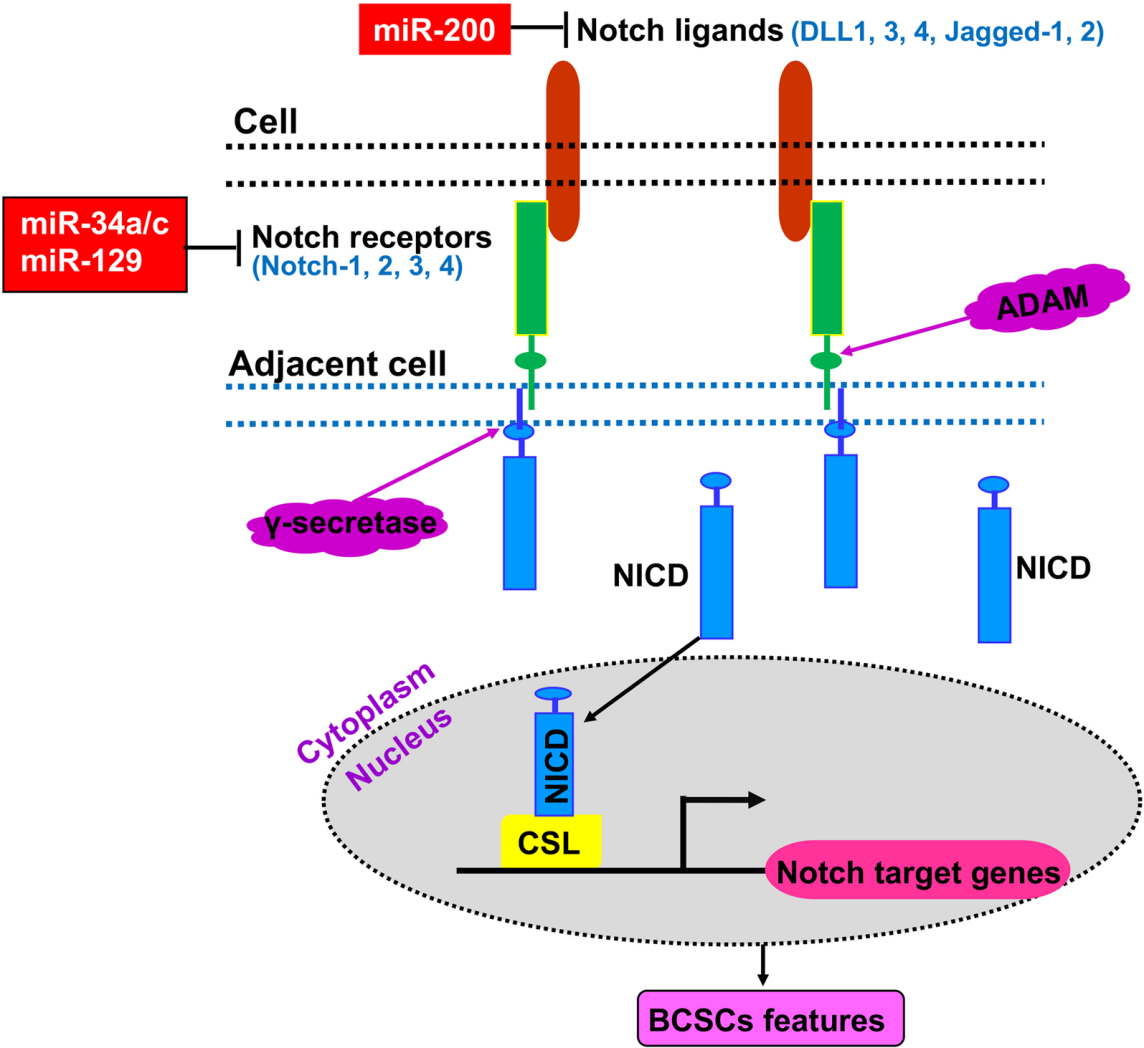
miRNA-mediated regulation of BCSCs via targeting the Notch signaling pathway. The Notch signaling pathway is activated when Notch ligands on one cell interact with Notch receptors on an adjacent cell, resulting in the two consecutive cleavages of Notch receptors by ADAMs and γ-secretase, and the release of Notch receptor intracellular domain NICD into the cytoplasm. The released NICD is translocated into the nucleus and binds to CSL to initiate the Notch target genes’ expression. The immediate targets of related miRNAs are marked

The Notch signaling is a primordial, evolutionally conserved pathway closely associated with cancer stem cell maintenance [55]. Dysregulation of this pathway occurs frequently in different types of human cancers, including breast cancer [56]. The Notch signaling pathway consists of three components: Notch ligands (DLL1, DLL3, DLL4, Jagged-1 and Jagged-2), Notch receptors (Notch-1, 2, 3 and 4) and the DNA binding sequence CSL. When a Notch ligand on one cell interacts with a Notch receptor on an adjacent cell, it liberates a Notch receptor intracellular domain (NICD), which can be recognized by ADAMs (a disintegrin and metalloproteases) and γ-secretase, respectively. The liberated NICD is translocated into the nucleus and binds to CSL, leading to corresponding transcription of downstream genes [57] (Fig. 3).

The Notch signaling regulates the self-renewal of CSCs. Members of the miR-34 family are considered tumor-suppressors and are associated with multiple human cancers. Overexpressed miR-34a reduces BCSC stemness, chemoresistance to doxorubicin and inhibits tumor formation by directly inhibiting Notch1 [58]. Furthermore, miR-34a is reduced in BCSCs. Increased miR-34a expression suppresses the Notch signaling pathway and subsequently inhibits breast cancer cell proliferation, migration, invasion, chemoresistance to paclitaxel (PTX) and BCSC propagation [59]. Additionally, miR-34c, another miR-34 family member, is downregulated in breast tumor-initiating cells (BT-ICs, also known as BCSCs), while overexpression of miR-34c in BC cells dramatically inhibited EMT, migration and self-renewal of BT-ICs via silencing its target gene *Notch4* [60]. Therefore, the miR-34 family (miR-34a/c) could serve as a promising target for prevention and therapy of breast cancer. Similarly, miR-200 family miRNAs are downregulated in BCSCs, and they inhibit BCSCs functions probably through suppressing Notch signaling by targeting Notch pathway components such as JAG1 and the mastermind-like Notch co-activators Maml2 and Maml3 [42].

On the other hand, miR-129 inhibits breast cancer cell’s self-renewal by suppressing Let-7b expression through directly inhibiting Estrogen Receptor 1 (ESR1). The decreased Let-7b releases its targeted inhibition of NUMB homologue and blocks the Notch oncogenic signaling [61] (Fig. 3).

### Roles of miRNAs in the PI3K/AKT/mTOR signaling pathway

**Fig. 4:**
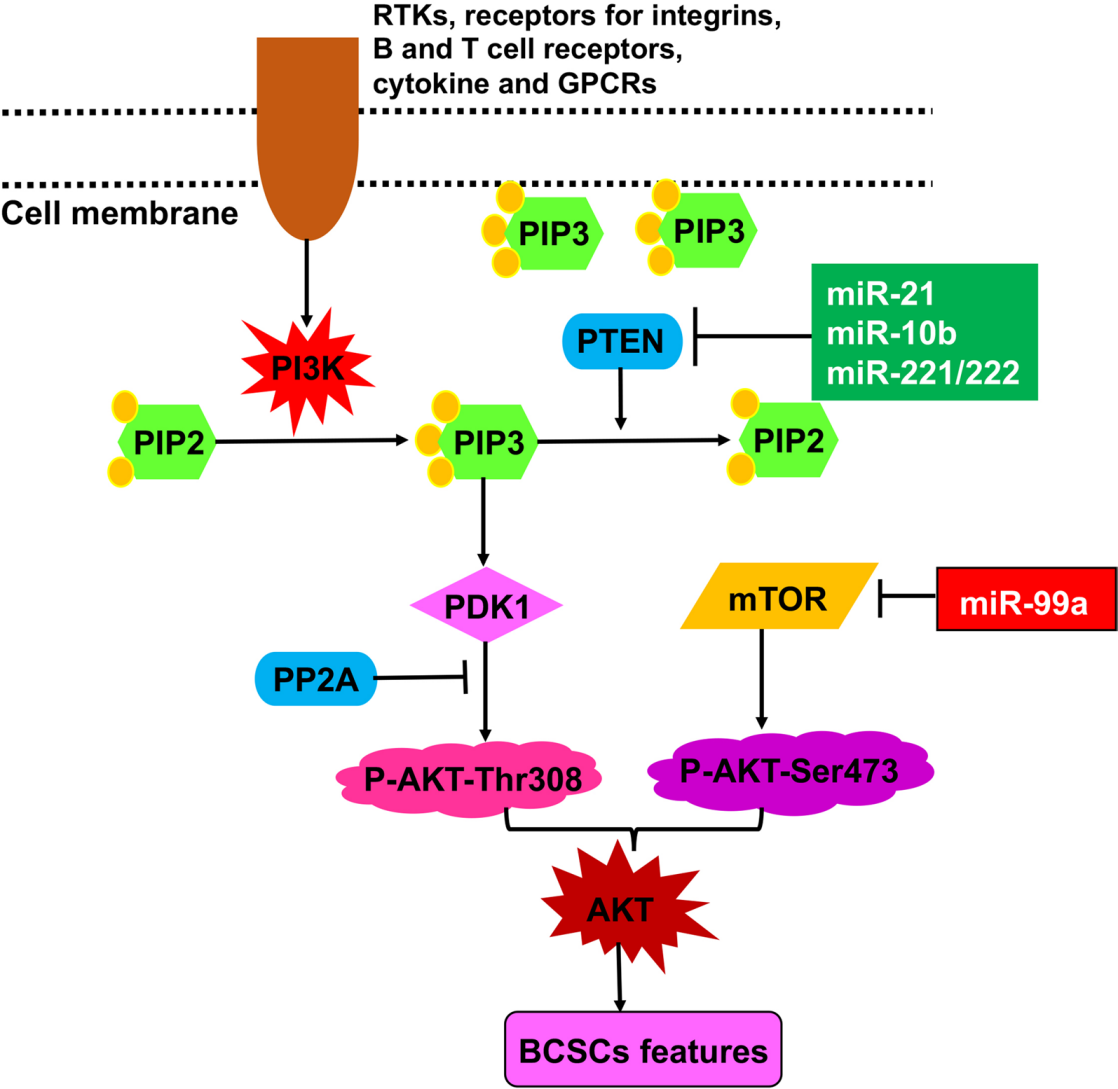
miRNA-mediated regulation of BCSCs via targeting the PI3K/AKT/mTOR signaling pathway. AKT is activated by various signals including RTKs, integrins, B and T cell receptors, cytokine receptors and GPCRs through PIP3 produced by PI3Ks. PIP3 interacts with the PH domain of AKT and recruits AKT to the cell membrane, allowing PDK1 to phosphorylate AKT-Thr308. mTOR acts both as a downstream effector and an activator of AKT by phosphorylating AKT-Ser473. Dually-phosphorylated AKT fully activates the PI3K/AKT/mTOR signaling pathway. The immediate targets of miRNAs are marked

The PI3K/AKT/mTOR signaling pathway is involved in the function and drug resistance of BCSCs [62]. Dysregulation of the PI3K/AKT/mTOR pathway are very common in many types of human cancers, including breast cancer [63]. Phosphatidylinositol 3-kinases (PI3K) can be activated by receptor tyrosine kinases (RTKs), integrins, B and T cell receptors, cytokine receptors and GPCRs, leading to phosphatidylinositol 4, 5 bisphosphate (PIP2) to be phosphorylated to phosphatidylinositol 3, 4, 5 trisphosphate (PIP3) and the generation of PIP3 in the plasma membrane. PIP3 then interacts with the PH domain of AKT and recruits AKT to the cell membrane. Next, phosphoinositide-dependent kinase-1 (PDK1) phosphorylates AKT at the Thr308 residue and activates AKT [64]. PTEN (Phosphatase and tensin homolog) dephosphorylates PIP3 to form PIP2, and is the most important negative regulator of AKT and an antagonist of PI3K. Mammalian target of rapamycin (mTOR) is not only a downstream member but also an activator of AKT; it can phosphorylate AKT at Ser473, facilitate its Thr308 phosphorylation by PDK1 and fully activate AKT [65] (Fig. 4). Recently, some studies reported that miRNAs regulate cancer progression through this pathway [66, 67] (Fig. 4).

miR-99a is found downregulated in a population of breast cancer stem-like cells known as SP cells. miR-99a suppresses SP self-renewal and tumorigenicity in vivo, and migration and invasion in vitro via suppressing the expression of *mTOR* [68], suggesting that miR-99a directly represses PI3K/AKT/mTOR signaling and reverses the phenotypes of BCSCs.

On the other hand, several miRNAs have been found to activate the PI3K/AKT signaling pathway through targeting PTEN. For example, miR-10b increases expression of stemness and EMT markers and promotes the self-renewal of BCSCs through repressing the transcription of *PTEN* [69]. Similarly, ectopic expression of miR-221/222 promotes breast cancer cell proliferation, migration, invasion, enriched proportion of CD44(+)/CD24(-) BCSCs and improves mammosphere formation capacity via downregulating PTEN [70]. Finally, antagomir-mediated interference of miR-21 activates PTEN expression, and thus reverses EMT, reduces migration and invasion in breast cancer cells, and decreases BCSC self-renewal capacity and clonogenicity [71].

### Roles of miRNAs in the BMI-1 signaling pathway

**Fig. 5:**
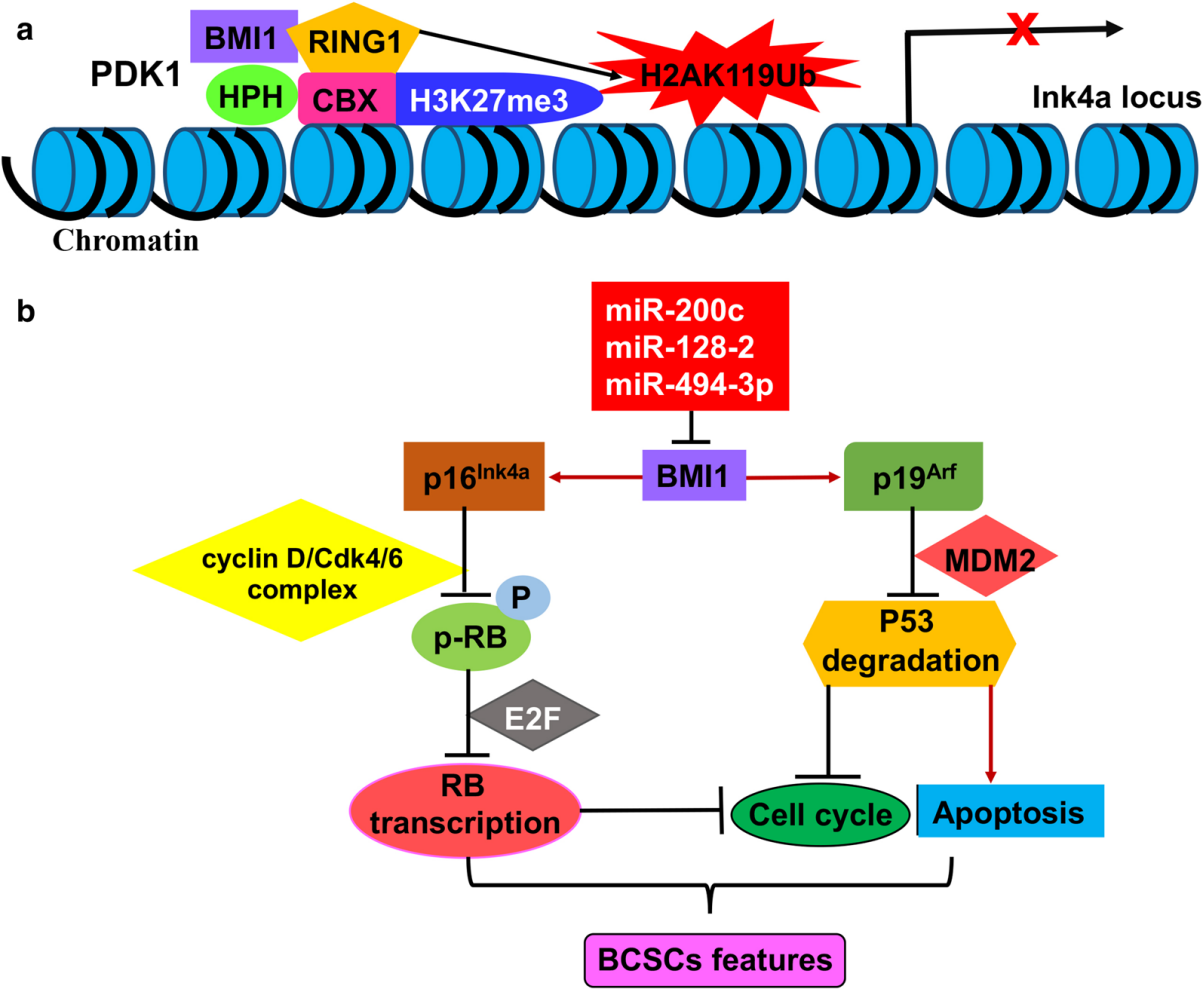
miRNA-mediated regulation of the BCSCs via targeting the BMI-1 signaling pathway. **a** To support self-renewal of stem cells by BMI-1 (a component of PRC1), PRC1 suppresses the expression of the Ink4a locus that encodes the *p16*^Ink4a^ and the *p19*^Arf^ genes by trimethylation of H3-K27 (H3K27me3) and ubiquitination of H2A-K119 (H2AK119Ub). The chromodomain of CBX binds to H3K27me3 and RING1 deposits monoubiquitin on H2AK119. **b** p16^Ink4a^ inhibits the phosphorylation of RB by the cyclin D/Cdk4/6 complex and E2F-dependent transcription of RB that finally inhibit cell cycle progression. p19^Arf^ causes high levels of p53 via preventing MDM2-mediated p53 degradation, which represses cell cycle progression and promotes apoptosis. The immediate targets of miRNAs are marked

BMI-1 (B lymphoma Mo-MLv insertion region 1 homolog) is a component of the polycomb repression complex 1 (PRC1). It acts as a self-renewal regulator to prevent senescence and apoptosis in normal and CSCs [72]. To support BMI-1-mediated self-renewal, PRC1 suppresses the Ink4a locus that encodes *p16*^Ink4a^ and *p19*^Arf^ genes by trimethylation of H3-K27 (H3K27me3) and ubiquitination of H2A-K119 (H2AK119Ub). Moreover, *p16*^Ink4a^ and *p19*^Arf^ are regulators of immortalization and senescence. Deletion and mutations of these two genes are frequently found in human cancers. The deubiquitinating enzyme USP16 suppresses self-renewal and senescence pathways in multiple tissues via deubiquitinating H2AK119 and increasing Ink4a locus transcription [73], while BMI-1 regulates cell cycle, apoptosis and senescence via inhibiting *p16*^Ink4a^ and *p19*^Arf^ genes encoded by *Ink4a* [74, 75]. BMI-1 is found to be upregulated in various human BC cells and is essential for self-renewal of BCSCs via suppressing genes involved in apoptosis and senescence [76] (Fig. 5).

The expression of *BMI-1* is modulated by some miRNAs in human cancers [77–79] (Fig. 5). For instance, miR-200b, miR-200c, miR-183 and miR-141 are dramatically downregulated in normal mammary stem cells and BCSCs, suggesting that the miR-200 family members play vital roles in regulating the self-renewal of BCSCs and normal mammary stem cells. Via downregulating BMI-1, miR-200c inhibits BC cell growth, induces cell differentiation and suppresses tumor formation in vivo. More importantly, it strongly suppresses the ability of normal mammary stem cells to form mammary ducts and suppresses tumor formation driven by human BCSCs in vivo [80, 81].

On the other hand, ectopic expression of miR-128 in BCSCs suppresses breast cancer progression and induces apoptosis by downregulating BMI-1 and ABCC5 expression [82]. In addition, the tropolone-related compound Hinokitiol is known to have an anti-cancer effect [83, 84]. It was found that Hinokitiol upregulates miR-494-3p to repress self-renewal of BCSCs via inhibiting BMI-1 expression [85].

### Roles of miRNAs in the STAT3 signaling pathway

**Fig. 6:**
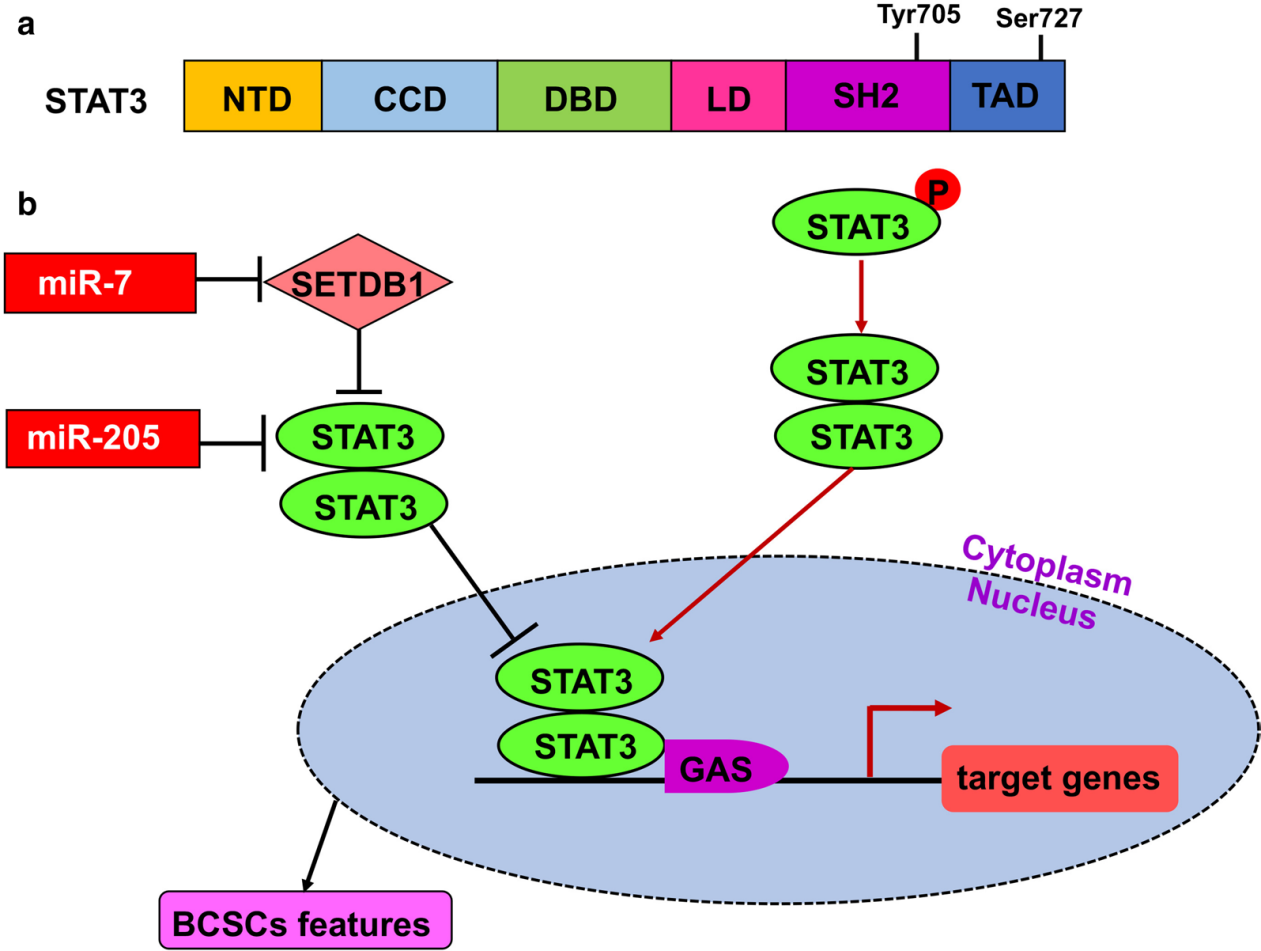
miRNA-mediated regulation of BCSCs via targeting the STAT3 signaling pathway. **a** STAT3 has six functional domains and two phosphorylation sites, as indicated. **b** STAT3 is mainly activated by Tyr705 phosphorylation within the SH2 domain, which leads to its dimerization. Activated STAT3 dimers interact with importins, translocate into the nucleus and activate target gene transcription by binding to the GAS motif of target genes. The immediate targets of miRNAs are marked

Signal Transducer and Activator of Transcription 3 (STAT3) is not only a transcription factor that plays a vital role in the biology of transformed and normal cells but also an important regulator of normal and BCSCs. It has six functional domains, including an N-terminal domain (NTD), a coiled coil domain (CCD) that mediates protein-protein interactions, a DNA-binding domain (DBD), a linker domain (LD), an SH2 (SRC homology 2) domain and a C-terminal transcription activation domain (TAD). It also has two important phosphorylation sites: Tyr705 within the SH2 domain, and Ser727 within TAD [86, 87]. STAT3 is mainly activated by phosphorylation of the conserved Tyr705 residue, which leads to its dimerization [88]. The activated STAT3 dimers interact with importins, translocate into the nucleus and activate target genes by binding to their GAS (interferon-γ-activated sequence) motifs [86, 89–91] (Fig. 6).

Several studies revealed that miRNAs are critical regulators of STAT3 in many types of human cancers [92] (Fig. 6). For instance, miR-7 inhibits metastasis and population growth, reverses EMT of BCSCs via directly suppressing oncogene *SETDB1.* Since SETDB1 transcriptionally activates the expression of STAT3, miR-7-mediated *SETDB1* repression downregulates the STAT3 signaling pathway [93]. miR-205 is thought to be not only a tumor suppressor but also an oncomiR in breast cancer [94]. As a suppressor, ectopic expression of miR-205 inhibits cell anchorage-independent growth and proliferation by repressing expression of ErbB3, which is a component of the most potent oncogenic complex ErbB2/ErbB3 heterodimer in breast cancer [95]. Moreover, miR-205 inhibits breast cancer invasion by directly targeting the key angiogenesis regulator VEGF-A [95]. As an oncomiR, miR-205 promotes the colony-forming potential of mammary epithelial cells and leads to an expansion of the progenitor-cell population by targeting the tumor suppressor gene phosphatase and tensin homolog (PTEN) [96]. It also has been shown to play an important role in the maintenance of BCSCs via inhibiting CSC self-renewal capacity and suppressing expression of stem cell markers CD44, ALDH1, TAZ and E2Q-E12, most likely through regulating STAT3 signaling [97].

## Conclusion and future perspective

It is now well-established that breast cancer harbors a small population of BCSCs, which are considered major oncogenic driving cells, as they facilitate breast tumor metastasis, relapse, progression and confer breast cancer therapy resistance by bestowing abilities such as migration, invasion, sphere formation, pluripotency, low-proliferation rate, self-renewal and tumor-initiation [4]. Hence, they play important roles in the resistance to therapeutic agents and the development of tumor heterogeneity by contributing to the propagation of neoplastic cells.

Although multiple mechanisms have been associated with stemness maintenance and BCSC functions attributed to the oncogenic potential, there are still unanswered questions calling for further investigation. Recent development in breast cancer research has revealed that miRNAs are promising therapeutic targets for breast cancer via their driving oncogenic potential and therapeutic hindrance due to BCSCs. Indeed, they are deregulated in various pathological conditions including CSCs during cancer progression.

In this review, we summarized recent advances for the roles of various miRNAs in a few BCSC-related signaling pathways important for maintenance of CSCs during breast cancer progression. Hence, targeting miRNAs is central for the elimination of BCSCs and breast cancer recurrence, therapeutic resistance and metastasis as miRNAs play regulatory roles in various characteristics of BCSCs.

In the future, therapeutic potentials could be further explored in breast cancer cells expressing an aberrant level of miRNAs. Several miRNA-based analogs and antagonists are currently being investigated and under clinical trials. For example, MRX34 (Mirna Therapeutics, Inc., Austin, TX, USA) is a liposomal product designed to deliver an analog of the naturally occurring tumor suppressor miR-34 [98], whereas most miRNAs are still explored at subclinical animal experimental levels and their therapeutics are still in infancy. Taken together, remarkable development and efforts are needed to bring the miRNA-based therapy from labs to the clinic.

## Data Availability

Not applicable.
